# Overexpression of PTEN suppresses lipopolysaccharide-induced lung fibroblast proliferation, differentiation and collagen secretion through inhibition of the PI3-K-Akt-GSK3beta pathway

**DOI:** 10.1186/2045-3701-4-2

**Published:** 2014-01-06

**Authors:** Zhengyu He, Yuxiao Deng, Wen Li, Yongming Chen, Shunpeng Xing, Xianyuan Zhao, Jia Ding, Yuan Gao, Xiangrui Wang

**Affiliations:** 1 Department of Anesthesiology, Ren Ji Hospital, School of Medicine, Shanghai Jiao Tong University, 1630 Dong Fang Road, Shanghai 200127, China

**Keywords:** Lung fibroblasts, Proliferation, Collagen, Lipopolysaccharide, Phosphoinositide3-kinase-Akt pathway, Glycogen synthase kinase 3beta, Phosphatase and tensin homolog

## Abstract

**Background:**

Abnormal and uncontrolled proliferation of lung fibroblasts may contribute to pulmonary fibrosis. Lipopolysaccharide (LPS) can induce fibroblast proliferation and differentiation through activation of phosphoinositide3-Kinase (PI3-K) pathway. However, the detail mechanism by which LPS contributes to the development of lung fibrosis is not clearly understood. To investigate the role of phosphatase and tensin homolog (PTEN), a PI3-K pathway suppressor, on LPS-induced lung fibroblast proliferation, differentiation, collagen secretion and activation of PI3-K, we transfected PTEN overexpression lentivirus into cultured mouse lung fibroblasts with or without LPS treatment to evaluate proliferation by MTT and Flow cytometry assays. Expression of PTEN, alpha-smooth muscle actin (alpha-SMA), glycogen synthase kinase 3 beta (GSK3beta) and phosphorylation of Akt were determined by Western-blot or real-time RT-PCR assays. The PTEN phosphorylation activity was measured by a malachite green-based assay. The content of C-terminal propeptide of type I procollagen (PICP) in cell culture supernatants was examined by ELISA.

**Results:**

We found that overexpression of PTEN effectively increased expression and phosphatase activity of PTEN, and concomitantly inhibited LPS-induced fibroblast proliferation, differentiation and collagen secretion. Phosphorylation of Akt and GSK3beta protein expression levels in the LPS-induced PTEN overexpression transfected cells were significantly lower than those in the LPS-induced non-transfected cells, which can be reversed by the PTEN inhibitor, bpV(phen).

**Conclusions:**

Collectively, our results show that overexpression and induced phosphatase activity of PTEN inhibits LPS-induced lung fibroblast proliferation, differentiation and collagen secretion through inactivation of PI3-K-Akt-GSK3beta signaling pathways, which can be abrogated by a selective PTEN inhibitor. Thus, expression and phosphatase activity of PTEN could be a potential therapeutic target for LPS-induced pulmonary fibrosis. Compared with PTEN expression level, phosphatase activity of PTEN is more crucial in affecting lung fibroblast proliferation, differentiation and collagen secretion.

## Background

Various acute lung injuries (ALI) can develop into acute respiratory distress syndrome (ARDS) with diffuse pulmonary fibrosis [1-3], which may result in respiratory failure [4]. Occurrence of ALI and ARDS can be due to exposure to lipopolysaccharides (LPSs), endotoxins produced by Gram-negative bacteria. Previous studies have found that focal aggregation of lung fibroblasts occurred prior to formation of fibrosis [5], implying that aberrant proliferation of fibroblasts takes place in the early stages of ALI/ARDS. Pulmonary fibrosis is characterized by fibroblast proliferation and differentiation to myofibroblast that are responsible for production of collagen [6,7]. Our previous studies have shown that LPS was able to directly induce secretion of collagen in primary cultured mouse lung fibroblasts *via* Toll-like receptor 4 (TLR4)-mediated activation of the phosphoinositide3-kinase-Akt (PI3-K-Akt) pathway [8,9]. LPS was also reported to induce fibroblasts proliferation [10], down-regulate phosphatase and tensin homolog (PTEN) expression [11,12].

The *PTEN* gene is recognized as a tumor suppressor with dephosphorylation activity [13]. Downregulation of PTEN expression and suppression of its dephosphorylation activity induce proliferation and inhibit apoptosis of glioma cells through activation of the PI3-K-Akt-glycogen synthase kinase 3 (GSK3) pathway, suggesting that PTEN may be involved in inactivation of PI3-K signaling [14]. PTEN restoration was also related to the inhibition of differentiation of human lung fibroblasts into myofibroblasts through extracellular signal-related kinase (ERK)/Akt inhibition [15]. The negative regulatory role of PTEN on the PI3-K-Akt pathway suggests that, without LPS stimulation, PTEN prevents the proliferation of lung fibroblasts, and that overexpression of PTEN might abrogate the fibroblast proliferation, differentiation, activation of PI3-K-Akt-GSK3β and collagen secretion induced by LPS. Thus, the mechanism by which PTEN is directly involved in LPS-induced fibroblast proliferation through regulation of the PI3-K-Akt-GSK3β pathway requires further elucidation.

In the present study we investigated the role of PTEN in LPS-induced lung fibroblast proliferation differentiation and collagen secretion, and explored the potential mechanism by which overexpression of PTEN inhibits LPS-induced lung fibroblast proliferation, differentiation, activation of PI3-K-Akt-GSK3 pathways and collagen secretion.

## Results

### PTEN expression and dephosphorylation activity in mouse lung fibroblasts transfected with Pten overexpression lentivirus

In the *Pten*-transfected primary cultured mouse lung fibroblasts, overexpression of PTEN and changes in PTEN dephosphorylation activity was detected by measuring *Pten* mRNA through real-time PCR and PTEN protein via Western blot. Malachite green-based assay was used to measure the PTEN dephosphorylation activity.

**Figure 1:**
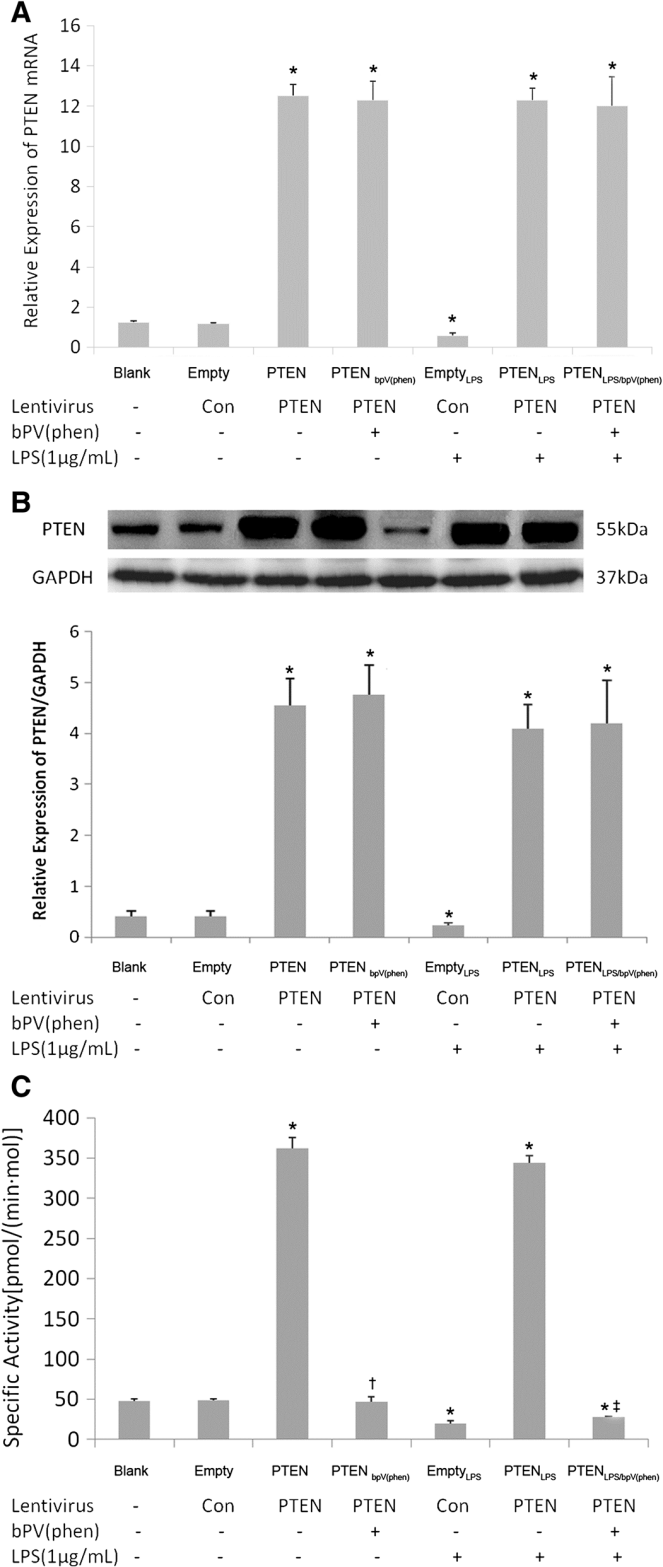
**Expression and dephosphorylation activity of PTEN in lung fibroblasts transfected with PTEN overexpression lentivirus.** The total RNA and cellular protein were collected from lung fibroblasts transfected with PTEN overexpression lentivirus (5 × 10^4^ TU/mL) for 48 h and treated with bpV(phen)(1 μM) for 0.5 h before exposure of the cells to 1 μg/mL LPS for up to 72 h, followed by detecting PTEN mRNA, protein expression and dephosphorylation activity by real-time RT-PCR **(1A)**, Western blot **(1B)** and Malachite green-based assay **(1C)**. *p < 0.05 *vs.* Blank and Empty group; ^†^*p* < 0.05 *vs.* PTEN group; ^‡^*p* < 0.05 *vs.* PTEN_LPS_ group. Columns represent mean values and error bars represent SD. Blots are representative of three independent experiments.

Levels of *Pten* mRNA and PTEN protein, and the dephosphorylation activity of PTEN, were significantly reduced in the Empty_LPS_ group (cells transfected with the empty vector and treated with LPS), compared with the cells transfected with the empty vector but without LPS (Empty group). These levels were significantly increased in the PTEN_LPS_ group (*Pten*-transfected cells treated with LPS) 72 h after LPS challenge (*p* < 0.05), compared to the Empty_LPS_ group. This indicates that LPS inhibited PTEN expression in non-transfected control cells, and that the *PTEN* lentiviral overexpression vector effectively increased PTEN expression in the transfected primary mouse lung fibroblasts (Figure 1A).

In *Pten*-transfected cells treated with LPS, treatment with the PTEN inhibitor 1 μM bpV(phen) 72 h after the LPS challenge(PTEN_LPS/bpV(phen)_ group) significantly reduced PTEN dephosphorylation activity, but had no effect on *Pten* mRNA and PTEN protein expression levels, compared to *Pten*-transfected cells treated with LPS but without the PTEN inhibitor(PTEN_LPS_ group, Figure 1B, C). This shows that bpV(phen) inhibited PTEN dephosphorylation activity, but had no effect on mRNA and protein expression.

### Effect of PTEN overexpression on activation of PI3-K-Akt-GSK3β pathway

**Figure 2:**
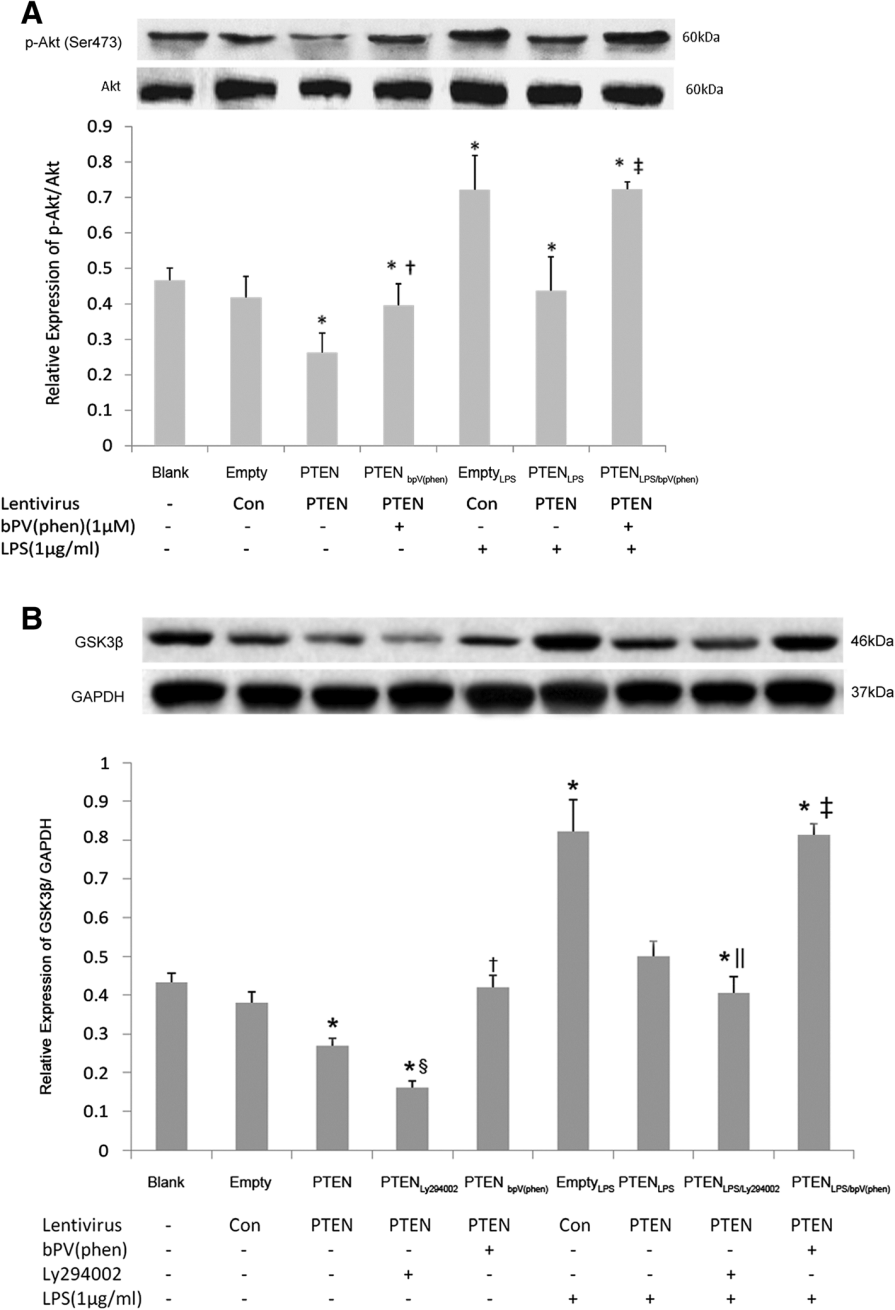
**The effect of PTEN overexpression on activation of PI3-K-Akt-GSK3β pathway in lung fibroblasts.** Cellular protein was collected from lung fibroblasts treated with 1 μM bpV(phen) for 0.5 h before exposure of the cells to LPS and transfected with PTEN overexpression vector for up to 72 h. Afterwards, the total and phosphor-Akt (Ser473) **(2A)** and GSK3β **(2B)** were detected by Western Blot. *p < 0.05 *vs.* Blank and Empty group; ^†^*p* < 0.05 *vs.* PTEN group; ^‡^*p* < 0.05 *vs.* PTEN_LPS_ group. ^§^*p* < 0.05 vs. PTEN group; ^||^*p* < 0.05 *vs.* PTEN_LPS_ group. Columns represent mean values and error bars represent SD. Blots are representative of three independent experiments.

To explore the detail mechanism underlying the effect of PTEN activity on LPS-induced lung fibroblast proliferation, activation of PI3-K-Akt-GSK3β and collagen secretion, we next tested the role of PTEN on activation of the PI3-K-Akt-GSK3β pathway in the LPS-induced fibroblast proliferation as assessed by Western blot. Compared to groups that were not treated with LPS (i.e., transfected with the empty vector [Empty group] or *Pten*-transfected but given no other treatments [PTEN group]), cells of the Empty_LPS_ group (transfected with the empty vector and treated with LPS) showed a significant increase in phosphorylation of Akt (Ser473) and GSK3β expression 72 h after LPS treatment (*p* < 0.05, both). Therefore, treatment with LPS increased Akt phosphorylation and GSK3β expression. However, in the *Pten*-transfected cells treated with LPS (PTEN_LPS_ group), the phosphorylation of Akt and GSK3β expression was significantly reduced compared with LPS-treated cells that were transfected with the empty vector (Empty_LPS_ group, *p* < 0.05), and was comparable to groups that were not given the LPS treatment (i.e., *Pten*-transfected [PTEN group] or transfected with an empty vector [Empty group], *p* > 0.05; Figure 2A-B). Thus, the overexpression of PTEN abrogated the effect of the LPS.

Most notably, in the *Pten*-transfected cells treated with LPS and the PTEN inhibitor bpV(phen)(PTEN_LPS/bpV(phen)_ group), phosphorylation of Akt and GSK3β expression was significantly increased 72 h after LPS treatment, compared with those given the same treatments but without bpV(phen)( PTEN_LPS_ group), and in fact was no different from the cells transfected with the empty vector and treated with LPS (Empty_LPS_ group, *p* > 0.05; Figure 2A-B).

In addition, we showed that treatment of Ly294002, the specific PI3-K-Akt inhibitor, in *Pten*-transfected cells could enhance the inhibition effect of PTEN on GSK3β expression with or without LPS treatment (PTEN group *vs.* PTEN_Ly294002_ group or PTEN_LPS_ group *vs.* PTEN_LPS/Ly294002_ group, *p* < 0.05; Figure 2B). This further demonstrated that downregulation of GSK3β was induced through inhibition of PI3-K-Akt pathway.

Collectively, these results above indicated that overexpression of PTEN inhibited LPS-induced lung fibroblast proliferation by inhibiting PI3-K-Akt-GSK3β pathway.

### Effect of PTEN overexpression on LPS-induced fibroblast proliferation

**Figure 3:**
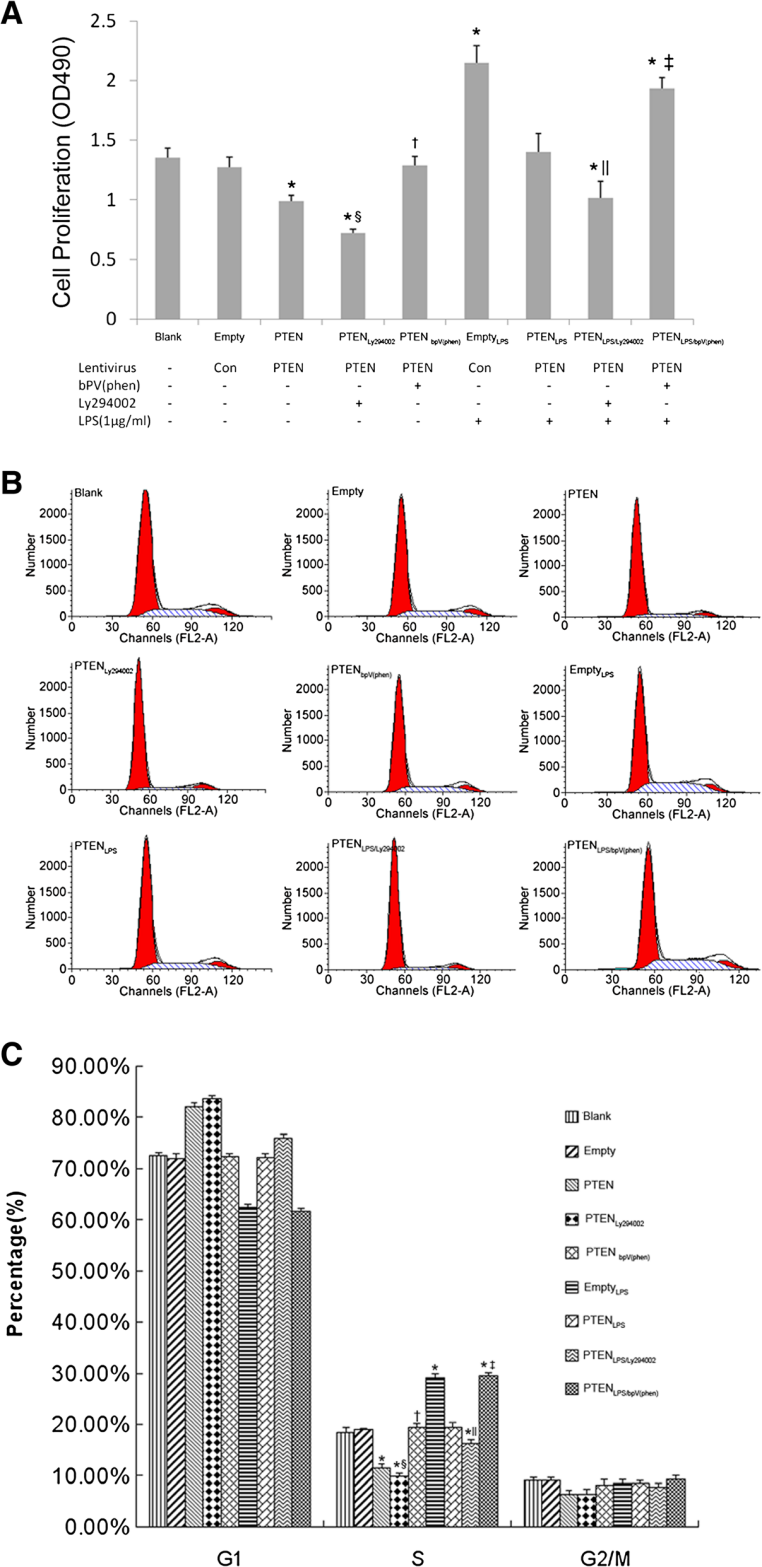
**The effect of PTEN overexpression on proliferation of LPS-induced lung fibroblasts.** The effect of overexpression of PTEN on lung fibroblast proliferation at 72 h after 1 μg/mL LPS challenge was detected using MTT and Flow cytometry assays (**3A**, MTT assay. **3B** and **3C**, Flow cytometry assay). bpV(phen)(1 μM for 0.5 h) was examined to investigate the effect of overexpression of PTEN on lung fibroblast proliferation. PI3-K inhibitor Ly294002 (50 μmol/L for 1 h) was used to assess the effect of PTEN overexpression and PI3-K-Akt pathway inhibition on lung fibroblast proliferation in the presence or absence of LPS. *p < 0.05 *vs.* Blank and Empty group; ^†^*p* < 0.05 *vs.* PTEN group; ^‡^*p* < 0.05 *vs.* PTEN_LPS_ group. ^§^*p* < 0.05 vs. PTEN group; ^||^*p* < 0.05 *vs.* PTEN_LPS_ group. Columns represented mean values and error bars represented SD. Flow cytometry graphs shown in Figure **B** were representative of three independent experiments.

To investigate the effect of PTEN overexpression on LPS-induced fibroblast proliferation, the MTT assay and flow cytometry were performed. Our results showed that, compared to the cells that were not *Pten*-transfected (Empty group), cell proliferation (viability) and the number of cells in S phase were significantly higher in those treated with LPS (Empty_LPS_ group), 72 h after treatment (*p* < 0.05; Figure 3A-C). However, in the *Pten*-transfected cells treated with LPS (PTEN_LPS_ group), cell proliferation (viability) and the S phase cell ratio was significantly reduced 72 h after LPS was administered, compared with the LPS-treated cells transfected with the empty vector (Empty_LPS_ group), but was virtually the same as both the *Pten*-transfected and empty vector-transfected cells that were not treated with the LPS (PTEN group and Empty group, *p* > 0.05; Figure 3A-C).

In *Pten*-transfected cells treated with LPS and the PTEN inhibitor bpV(phen) (PTEN_LPS/bpV(phen)_ group), cell proliferation (viability) and the S phase cell ratio were significantly greater after bpV(phen) was given 72 h after LPS treatment, compared with identically treated cells that did not receive PTEN inhibitor (PTEN_LPS_ group). However, these amounts were similar to those of the cells transfected with the empty vector and treated with LPS (Empty_LPS_ group, *p* > 0.05; Figure 3A-C).

In comparisons between *Pten*-transfected cells treated or not with the specific PI3-K-Akt inhibitor Ly294002, it was found that application of Ly294002 significantly decreased cell proliferation (viability) and the S phase cell ratio of lung fibroblasts. This significant decrease was also shown between *Pten*-transfected cells treated with LPS, with or without Ly294002 (PTEN_Ly294002_ group *vs.* PTEN_LPS/Ly294002_ group; Figure 3A-C). The above results are strong evidence that the expression and activity of PTEN has an important role in the inhibition of LPS-induced fibroblast proliferation.

### Effect of PTEN overexpression on LPS-induced fibroblast differentiation and collagen secretion

To investigate the effect of PTEN overexpression on LPS-induced fibroblast differentiation and collagen secretion, the expression of alpha smooth muscle actin (α-SMA), the symbol of lung fibroblast-to-myofibroblast differentiation [6], were detected by Western blot; And the content of C-terminal propeptide of type I procollagen (PICP), a segment degraded from the C-terminal by the procollagen C-endopeptidase and a marker of type I collagen secretion [16], in cell culture supernatants was examined by ELISA.

**Figure 4:**
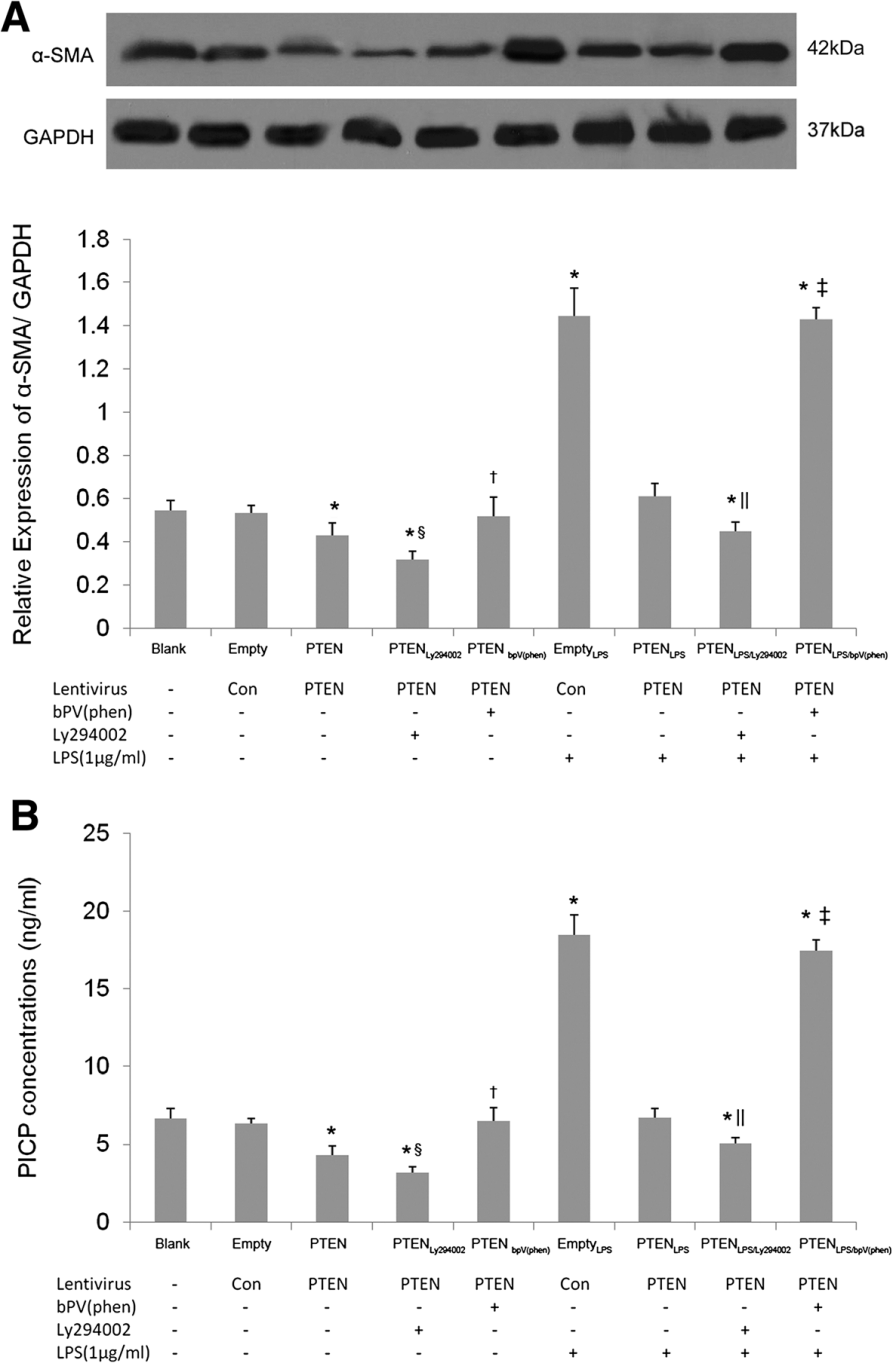
**The effect of PTEN overexpression on differentiation and collagen secretion of LPS-induced lung fibroblasts.** Cellular expression of α-SMA examined by Western blot **(A)** and PICP content in cell culture supernatants detected by ELISA **(B)** were used to reflect the effect of overexpression of PTEN on lung fibroblast differentiation and collagen secretion 72 h after 1 μg/mL LPS challenge. bpV(phen)(1 μM for 0.5 h) was used to investigate the effect of overexpression of PTEN on lung fibroblast differentiation and collagen secretion. PI3-K inhibitor Ly294002 (50 μmol/L for 1 h) was used to assess the effect of PTEN overexpression and PI3-K-Akt pathway inhibition on lung fibroblast differentiation and collagen secretion in the presence or absence of LPS. *p < 0.05 *vs.* untreated control group; ^†^*p* < 0.05 *vs.* PTEN group; ^‡^*p* < 0.05 *vs.* PTEN_LPS_ group. ^§^*p* < 0.05 vs. PTEN group; ^||^*p* < 0.05 *vs.* PTEN_LPS_ group. Columns represent mean values and error bars represent SD (Mean±SD). Blots are representative of three independent experiments.

Similar to PTEN overexpression on LPS-induced fibroblast proliferation, LPS treatment could increase the expression of α-SMA in lung fibroblast and levels of PICP in cell culture supernatants, which could be overcame by PTEN overexpression. The application of Ly294002 aggravated the inhibition effect of PTEN, while the treatment of bpV(phen) overcome this (p < 0.05, Figure 4A-B).

## Discussion

It is generally accepted that LPS-induced pulmonary fibrosis involves the proliferation and differentiation of lung fibroblasts [17,18]. PTEN, a tumor suppressor, is involved in the proliferation of various cells [19-23]; a decrease in PTEN expression results in the activation of the PI3-K-Akt signaling pathway [24]. Therefore, further study exploring the mechanism by which PTEN influences LPS-induced lung fibroblast proliferation and differentiation has important clinical implications. Our results in the present study indicate that LPS-induced downregulation of PTEN is directly involved in fibroblast proliferation, differentiation and collagen secretion by way of the PI3-K-Akt-GSK3β pathway, and could be overcome by the overexpression of PTEN. This suggests that PTEN may be a potential intervention target for pulmonary fibrosis.

A mutation or deletion in *PTEN* have been confirmed to affect various cell biological behaviors [25,26] including proliferation [19-23] collagen metabolism [27] and oncogenesis [28]. In our study, PTEN expression and its dephosphorylation activity were inhibited when cells were stimulated with LPS; the underlying mechanism remains unclear but may be correlated with LPS-induced activation of transcription factors such as c-Jun, NF-κB, and HES-1 [24,29-31]. This needs to be studied further.

Previous studies have found that *PTEN* methylation [32] and its knockout through RNA interference [33] increased cell proliferation and collagen metabolism [27], as did dephosphorylation of its protein product [34]. Our results in the present study further showed that LPS-induced cell proliferation, differentiation and collagen secretion could be inhibited in lung fibroblasts transfected with a *PTEN* overexpression lentivirus, which increased both PTEN levels and its dephosphorylation activity. Similar results using a PEP-1-PTEN fusion protein transfected into macrophages [35] or adenovirus-mediated PTEN gene transferred into synovial fibroblasts [36] were reported. Therefore, we reasoned that a decrease in PTEN expression and its dephosphorylation activity could be directly involved in inhibiting LPS-induced lung fibroblast cell proliferation, differentiation and collagen secretion, and overexpression of PTEN may have potential for pulmonary fibrosis treatment. This finding would be strengthened if *in vivo* model, such as PTEN KO or transgenic mice, were used to further confirm this.

The loss of PTEN, activation of the PI3-K-Akt signaling pathway, or both is associated with cancer cell proliferation and metastasis [14,37-39]. Protein products of the *PTEN* gene can inactivate PI3-K activity with its dephosphorylation activity [40]. We previously showed that blockade of PI3-K using a pharmacological inhibitor (Ly294002) decreased lung fibroblast collagen secretion [41]. As a downstream molecule of PI3-K-Akt, GSK3β is also involved in cell growth and other cell cycle-related biological functions [42-44]. Activation or phosphorylation of GSK3β was found to be a factor in LPS-induced or TLR4-mediated pro-inflammatory cytokine production in immune cells [45-47]. In the current study, we found that overexpression of PTEN enhanced the inhibitory effect of Ly294002 on cell growth, differentiation and collagen secretion concomitant with suppression of phosphorylation of Akt. Our results also suggested that activation of GSK3β was involved in the LPS-induced lung fibroblast proliferation, differentiation and collagen secretion. Considering GSK3β was found to be an important downstream molecule of PI3-K-Akt in our previous studies [48] and that of others [49], we reasoned that the activation of PI3-K-Akt-GSK3β complex signaling pathways played important role in mediating the LPS-induced lung fibroblast proliferation, differentiation and collagen secretion.

Thus, we think that LPS could activate the PI3-K-Akt-GSK3β signaling pathway by inhibiting PTEN expression and dephosphorylation activity, thereby promoting fibroblast proliferation, differentiation and collagen secretion. In fact, we show that the PTEN inhibitor bpv(phen), which inhibited PTEN dephosphorylation activity and had no effect on its expression [50], overcame the effect of LPS. This suggests that expression of PTEN and PTEN dephosphorylation activity may have a causal association with the activity status of the PI3-K-Akt-GSK3β pathway during LPS-induced lung fibroblast proliferation, differentiation and collagen secretion.

Our present study showed that lentiviral-mediated PTEN overexpression inhibited activation of the PI3-K-Akt pathway and lung fibroblast proliferation, differentiation and collagen secretion, with or without LPS-stimulation. However, these changes could be reversed by treatment with the PTEN dephosphorylation activity inhibitor, bpv (phen). This implies that the dephosphorylation activity of PTEN is more crucial in the regulation of lung fibroblast functions than PTEN expression. These findings were in accord with one study using lung cancer cells [51]. More experiments using *PTEN* short interfering RNA (siRNA) are required to further confirm the role of PTEN in affecting lung fibroblast functions. In addition, whether LPS-induced Akt phosphorylation or GSK3β expression is the major cause of fibroblast proliferation needs to be determined. Other studies have shown that TSC-2 [52], PRAS40 [53], mTORC [54], GSK3 [55] and FOXO [56] are involved in the phosphorylation of Akt, cell proliferation, and survival pathways. Thus, further determining the role of Akt using Akt siRNA or GSK3β siRNA in lung fibroblast proliferation may be required.

In addition, Akt is also an important anti-apoptotic and pro-survival kinase during the cellular response to cell injury. It is possible that the inhibition of lung fibroblast proliferation is in part a consequence of increased cell apoptosis. But, we have not found any significant apoptotic changes in lung fibroblast after LPS treatment in present study (data not shown). Therefore, more experiments are needed to confirm this in the future.

## Conclusions

Collectively, we show that PTEN is an important negative regulator of pathogenesis of pulmonary fibrosis induced by LPS. Our extended work has confirmed that PTEN dephosphorylation activity and inactivation of the PI3-K-Akt-GSK3β signaling pathways are important in inhibiting the growth and differentiation of lung fibroblasts. Overexpression and induced phosphatase activity of PTEN inhibit LPS-induced lung fibroblast proliferation, differentiation and collagen secretion through inactivation of PI3K-Akt-GSK3β pathways; thus, expression and phosphatase activity of PTEN could be a potential therapeutic target for LPS-induced pulmonary fibrosis.

## Materials and methods

### Ethics statement

All procedures of this study were carried out in accordance with the guidelines for animal care published by the United States’ National Institutes of Health (NIH) for animal care (Guide for the Care and Use of Laboratory Animals, Department of Health and Human Services, NIH Publication No. 86–23, revised 1985).

### Primary cultures of mouse lung fibroblasts

Lung fibroblasts were isolated from a C57/BL6 mouse as described in our previous study [41]. Briefly, an eight-week-old mouse (Shanghai SLAC Laboratory Animal, China) was euthanized by decapitation. Lung tissues were promptly excised, washed with phosphate buffered saline (PBS), and cut to 1 mm^3^ pieces. The tissues were distributed evenly over the bottom of culture plates and covered with Dulbecco’s modified Eagle’s medium (DMEM) containing 10% calf serum (Gibco, USA). The plates were cultured at 37°C in a humidified 5% CO_2_ incubator (Labotect, Germany), and DMEM was changed every three days. When the cultures reached 80% confluence, adherent cells were detached by exposure to 0.25% trypsin for five minutes, and then passaged at a dilution of 1:4. Cells grew to a typical fusiform shape after four generations. Fibroblasts were characterized as previously described [57], and then used for the following experiments.

### Construction and identification of Pten overexpression lentivirus

A *Pten* overexpression lentivirus was constructed and verified by GeneChem (Shanghai, China). The *Pten* gene was amplified from a cDNA library via PCR (forward 3′-GAGGATCCCCGGGTACCGGTCGCCACCATGACAGCCATCATCAAAGAG-5′, reverse 5′-TCACCATGGTGGCGACCGGGACTTTTGTAATTTGTGAATGCTG-3′). *Pten* PCR products were inserted into the pGC-LV vector through restriction enzyme Age I linearization, and then the construction vector which carried the *Pten* gene fragment was transformed into DH5α competent cells. These were cultured at 37°C for 16 h. Positive clones containing the *Pten* coding sequence were selected by PCR. The vector containing the correct *Pten* sequence was named pGC-FV-Pten. In accordance with the method described by the GeneChem lentiviral operating manual, pGC-FV-Pten, pHelper 1.0, and pHelper 2.0 were transferred into 293 T cells. The recombinant virus was harvested and quantified.

### Experimental design and treatment

Purified mouse lung fibroblasts in DMEM containing 10% calf serum were seeded into 96-well plates and grown in a humidified atmosphere containing 5% CO_2_. When cells reached ~60% confluence, the medium was replaced with serum-free medium and the cultures were incubated for an additional 24 h at 37°C in 5% CO_2_. Finally, the serum-free medium was replaced with DMEM containing 10% calf serum and the cells were divided into several groups for various experimental manipulations as described below.

To augment levels of PTEN in the lung fibroblasts, the *Pten* overexpression lentivirus was added to cells at a concentration of 5 × 10^4^ transducing units (TU)/mL for 48 h prior to any other treatments. The PTEN_LPS_ group was then incubated with 1 μg/mL LPS (derived from O55:B5 *Escherichia coli*; Sigma, USA) for up to 72 h. To assess the effect of PTEN overexpression and PI3-K-Akt pathway inhibition on LPS-induced lung fibroblast proliferation, the *Pten*-transfected group PTEN_LPS/Ly294002_ was established by adding 50 μmol/L of the PI3-K inhibitor Ly294002 (CST, USA) to transfected cells for 1 h, followed by incubating with 1 μg/mL LPS for up to 72 h.

To inhibit the dephosphorylation activity of PTEN, *Pten*-transfected lung fibroblasts (PTEN_LPS/bpV(phen)_ group) were exposed to the PTEN inhibitor potassium bisperoxo (1,10-phenanthroline) oxovanadate (bpV[phen]; 1 μM; Alexis, USA) for 30 min. Afterwards, cells were incubated with 1 μg/mL LPS for up to 72 h.

Group PTEN consisted of transfected cells that were not given any other treatment. To establish group PTEN_Ly294002_, the transfected cells were treated with 50 μmol/L Ly294002 for 1 h without any other treatments. Group PTEN_bpV(phen)_ consisted of *Pten*-transfected cells that were given 1 μM bpV(phen) stimulation without LPS.

Negative controls were established by adding the same volume of control-lentivirus (i.e., containing no exogenous gene) for 48 h, and incubating the fibroblasts with (group Empty_LPS_) or without (group Empty) LPS for 72 h. Cells of group Blank received no treatments.

Experiments were performed in triplicate in each group. Cells were collected for measurements 72 h with or without LPS stimulation. Cell proliferation was assessed by the MTT assay and flow cytometry. The expressions of PTEN protein and phosphorylated Akt were examined by Western blot analysis. PTEN dephosphorylation activity was measured with a malachite green-based assay for inorganic phosphate [58].

### Real-time RT-PCR

The mRNA expression of *Pten* was analyzed via real-time RT-PCR. Total RNA was isolated from cells with an RNeasy kit using Trizol (Invitrogen, USA) and was reverse-transcribed into cDNA with a reverse transcription kit using M-MLV polymerase (Promega, USA). Sequence-specific primers were: glyceraldehyde 3-phosphate dehydrogenase (GAPDH)-F: 5′- TGGTGAAGGTCGGTGTGAAC-3′, GAPDH-R: 5′-GCTCCTGGAAGATGGTGATGG-3′; *Pten*-F: 5′-CCATAACCCACCACAGC-3′, *Pten* -R: 5′-AGTCCGTCCCTTTCCAG-3′. Real-time PCR was performed in an IQ5 PCR System (Bio-Rad, USA) with an initial denaturing step at 95°C for 15 s, 45 cycles of denaturing at 95°C for 5 s, and annealing at 60°C for 30 s. Relative expression of real-time PCR products was determined using the ΔΔCt method [59] to normalize target gene expression to that of the housekeeping gene (GAPDH).

### MTT assay

Cell proliferation was evaluated by a modified MTT assay. The test cells in exponential growth were plated at a final concentration of 2 × 10^3^ cells/well in 96-well culture plates for different culture time. MTT (10 μl, 10 mg/mL) was then added. After an additional 4 h of incubation, the reaction was terminated by removal of the supernatant and addition of 150 μl DMSO for 30 min. Optical density (OD) of each well was measured at 490 nm using ELISA reader (ELx808 Bio-Tek Instruments, USA).

### Flow cytometry assay

As an indicator of cell proliferation, Flow cytometry was performed to assess the relative percentages of cells at different phases in the cell cycle. Cells were harvested 72 h after LPS stimulation, fixed in 70% alcohol for 1 h at 4°C, permeabilized by incubation with PBS containing 0.2% Tween 20 at 37°C for 15 min, and incubated in PBS with 50 μg/mL propidium iodide (P4170, Sigma, USA) and 10 μg/mL RNase (EN0531, Fermentas, CA) for 1 h at 37°C. The fluorescence of 10^6^ cells was analyzed on BD FACSCalibur™ instruments (BD Biosciences, USA). G1, S, and G2/M ratios were calculated using CellQuest Pro Software (version 5.1, BD Biosciences, USA).

### Western blot analysis

Expressions of PTEN, Ser473 phospho-Akt, GSK3β and α-SMA were detected by Western blot. Briefly, cells were collected and lysed with 1 × RIPA lysis Buffer (50 mM Tris–HCl, pH 7.4, 150 mM NaCl, 1% Nonidet P-40, 0.5% deoxycholic acid, 0.1% sodium dodecyl sulfate [SDS], 5 mM ethylenediaminetetraacetic acid [EDTA], 2 mM phenylmethylsulfonyl fluoride [PMSF], 20 μg/mL aprotinin, 20 μg/mL leupeptin, 10 μg/mL pepstanin A, and 150 mM benzamidine) on ice for 10–15 min. Cell debris was pelleted by centrifugation, and protein-containing supernatants were collected. Protein quantification was performed with the bicinchoninic acid method, and SDS-polyacrylamide gel electrophoresis (PAGE) was performed. Proteins were transferred to polyvinylidene fluoride membranes, probed with the appropriate primary and secondary antibodies, and detected by the ECL + plus™ Western blotting system kit (Amersham, USA). Primary antibodies (1:1000 dilution) were: rabbit anti-phospho-Akt (Ser473; CST, USA), rabbit anti-Akt (CST, USA), rabbit anti-PTEN CST, USA), rabbit anti-phosphor-GSK3β (Ser9) (CST, USA), rabbit anti-α-SMA (Abcam, UK) and mouse anti-GAPDH (Santa Cruz Biotechnologies, USA). Secondary antibodies (1:5000) were: goat anti-mouse IgG (Santa Cruz Biotechnologies, USA) and goat anti-rabbit IgG (Santa Cruz Biotechnologies, USA). Immunoreactivity was visualized with Perfection 3490 photo gel imaging systems (Epson, Japan) and analyzed by Image Pro PLUS. Protein expression was normalized to GAPDH.

### Malachite green-based assay

The specific hydrolysis of phosphate at the 3 position on the inositol ring of diC16-phosphatidylinositol 3, 4, 5-triphosphate (PIP_3_) by PTEN was detected using a malachite green-based assay for inorganic phosphate. Reactions were carried out in a volume of 20 μL for various times at 37°C, then terminated by the addition of 20 μL of 0.1 M n-ethylmaleimide and 50 μL of malachite green reagent as described previously [58]. The absorbance at 620 nm was measured, and phosphate release quantified, by comparison to a standard curve of KH_2_ PO4 (1–1000 pM). Reactions were carried out in triplicate and the specific activities are represented as moles of phosphate released per min/per mole of enzyme, ± standard deviation (SD).

### ELISA of PICP

The concentration of PICP in cell culture supernatant, directly associated with type I procollagen synthesis, was measured by ELISA using mouse PICP ELISA kit (Biorbyt, UK.). All produces were carried out in accordance with operating instruction.

### Statistical analysis

All data are represented as mean ± SD. SPSS statistical software version 12.0 was used for mean value comparisons of single-factor multiple samples. The homogeneity of variance data were analyzed with the one-factor analysis of variance least squares difference test, and the heterogeneity of variance data were analyzed with the Kruskal Wallis rank sum test. *P-values* < 0.05 were considered statistically significant.

## Abbreviations

LPS: Lipopolysaccharide; PI3K: Phosphoinositide 3-kinase; PTEN: Phosphatase and tensin homolog; GSK3: Glycogen synthase kinase 3; bpV(phen): Potassium bisperoxo (1,10-phenanthroline) oxovanadate; GAPDH: Glyceraldehyde 3-phosphate dehydrogenase; α-SMA: Alpha smooth muscle actin; PICP: C-terminal propeptide of type I procollagen.

## Competing interests

The authors declare that they have no competing interests.

## Authors’ contributions

Conceived and designed the experiments: ZYH, XRW. Performed the experiments: ZYH, YG, YXD, WL, YMC, SPX. Analyzed the data: XYZ, JD. Wrote the paper: ZYH, YG, XRW. All authors read and approved the final manucript.
